# Managing the Planned Cessation of a Global Supply Market: Lessons Learned From the Global Cessation of the Trivalent Oral Poliovirus Vaccine Market

**DOI:** 10.1093/infdis/jiw571

**Published:** 2017-06-30

**Authors:** Jennifer Rubin, Ann Ottosen, Andisheh Ghazieh, Jacqueline Fournier-Caruana, Abraham Kofi Ntow, Alejandro Ramirez Gonzalez

**Affiliations:** 1 United National Children’s Fund (UNICEF) Supply Division, Copenhagen, Denmark; and; 2 World Health Organization, Geneva, Switzerland

**Keywords:** tOPV cessation, global vaccine market cessation, global supply management.

## Abstract

The Polio Eradication and Endgame Strategic Plan 2013-2018 calls for the phased withdrawal of OPV, beginning with the globally synchronized cessation of tOPV by mid 2016. From a global vaccine supply management perspective, the strategy provided two key challenges; (1) the planned cessation of a high volume vaccine market; and (2) the uncertainty of demand leading and timeline as total vaccine requirements were contingent on epidemiology. The withdrawal of trivalent OPV provided a number of useful lessons that could be applied for the final OPV cessation. If carefully planned for and based on a close collaboration between programme partners and manufacturers, the cessation of a supply market can be undertaken with a successful outcome for both parties. As financial risks to manufacturers increase even further with OPV cessation, early engagement from the cessation planning phase and consideration of production lead times will be critical to ensure sufficient supply throughout to achieve programmatic objectives. As the GPEI will need to rely on residual stocks including with manufacturers through to the last campaign to achieve its objectives, the GPEI should consider to decide on and communicate a suitable mechanism for co-sharing of financial risks or other financial arrangement for the outer years.

In May 2008, in line with guidance from the Strategic Advisory Group of Experts on Immunisation (SAGE), the World Health Assembly endorsed the principle of synchronized oral poliovirus vaccine (OPV) cessation globally. Recognizing that wild poliovirus type 2 was eradicated in 1999 and that >90% of circulating vaccine-derived poliovirus cases in recent years were caused by the vaccine-derived type 2 strain, in 2012 the SAGE further recommended the withdrawal of type 2 OPV (OPV2) as the first step toward complete withdrawal of all OPVs, the final date to be confirmed in October 2015 based on progress against predefined prerequisites. In May 2012, the 65th Resolution of the World Health Assembly declared polio eradication “a programmatic emergency for global public health,” requesting the World Health Organization to “undertake the development, scientific vetting, and rapid finalization of a comprehensive polio eradication and endgame strategy” and to inform member states of the potential timing of the switch from trivalent OPV (tOPV) to bivalent OPV (bOPV) in all routine immunization programs [1].

In 2013, the Global Polio Eradication Initiative (GPEI) published the *Polio Eradication and Endgame Strategic Plan 2013–2018* [2], which called for the phased withdrawal of OPV beginning with the globally synchronized cessation of use of OPV2 by mid 2016 [2, p 58, section 6.20], and final withdrawal in 2019–2020 [2, p 60]. The cessation was required to be synchronized globally across countries and regions to minimize risks of reintroducing type 2 vaccine viruses in unvaccinated populations. The final confirmation of cessation would take place at the SAGE meeting in October 2015. From a global vaccine supply perspective, the strategy provided 2 key challenges; (1) the planned global synchronized cessation of a high-volume vaccine market and (2) the uncertainty of demand leading up to cessation, given that the timeline and total vaccine requirements were contingent on epidemiological circumstances.

The purpose of the current article is to review the process used to plan for and implement the cessation of the tOPV market by the GPEI, identifying what worked well and what could be improved to inform planning and decision making for overall OPV cessation. The perspectives from the United Nations Children’s Fund (UNICEF) and other GPEI partners will be presented, in addition to feedback provided by vaccine suppliers. Because the review focuses on the global perspective, tOPV supply planning and management undertaken by self-producing and self-procuring countries is beyond its scope.

## TRIVALENT OPV CESSATION: A COMPLEX GLOBAL UNDERTAKING

**Figure 1.**
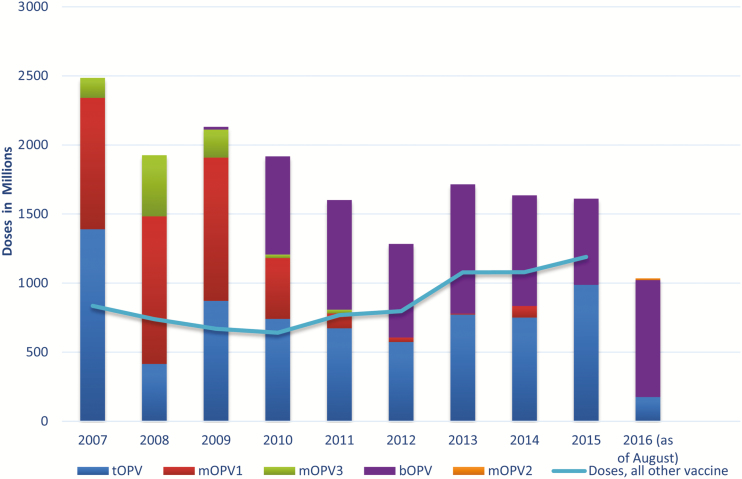
Procured quantities of oral poliovirus vaccine (OPV), 2007–2015—bivalent OPV (bOPV); monovalent OPV types 1, 2, and 3 (mOPV1, mOPV2, and mOPV3); and trivalent OPV (tOPV)

UNICEF is the procurement agency on behalf of the GPEI, responsible for securing sufficient supply of prequalified OPV for the supplementary immunization activity (SIA) calendar in about 50 countries, but it is also the procurement agency for >70 countries for routine immunization requirements based on bilateral agreements with these countries. The quantity of OPV that UNICEF procures annually is by far the highest compared with annual volumes of all other vaccines procured by UNICEF in 2007–2015 (Figure 1). Of the total vaccine doses procured through UNICEF in 2007, OPV made up 75%, declining to 58% in 2015. Annual OPV procurement through UNICEF peaked with 2.5 billion doses in 2007, when UNICEF also procured OPV for India, but it declined over the past 5 years to an annual average of about 1.5 billion doses. 

In 2016, UNICEF is projected to meet about 40% of global OPV demand outside of China (data presented by WHO during the WHO/UNICEF consultation with OPV and inactivated poliovirus vaccine manufacturers, 28 July 2016). Owing to the high annual volume of OPV, the workload to vaccine manufacturers—bulk production, filling, testing, packaging, and releasing—is extremely high, and changes in demand, including the prospects of market cessation, therefore have considerable implications for manufacturers’ resource allocations (eg, staff, investment planning, and maintenance), and such changes need to be planned for well in advance.

The global cessation of demand for tOPV and the simultaneous rollout of bOPV as a new vaccine in all routine immunization and SIAs that took place between the fourth quarter (Q4) of 2015 and the first quarter (Q1) of 2016, were unprecedented and complex undertakings representing a unique set of challenges for the GPEI, 155 OPV-using countries and territories, and vaccine manufacturers, requiring significant advance planning and coordination to ensure a smooth transition and protect the gains of the polio eradication program while minimizing financial risks to all stakeholders. Leading up to the switch, the key risk related to tOPV for the GPEI was to not have access to sufficient supply, including for planned precessation campaigns to boost immunity to type 2 poliovirus and thereby reduce risks of type 2 outbreaks after the switch. For countries, a key concern was to have enough stocks of tOPV while minimizing residual stocks at the time of the switch to minimize the costs of withdrawing and destroying doses. Similarly, from the vaccine manufacturers’ perspective, a key risk was to end up with considerable residual stocks of tOPV at the time of cessation, which would entail financial loss and write-off.

From the supply planning perspective, the challenges were aggravated by the fact that the cessation of the tOPV demand market was scheduled to be confirmed only 6 months before implementation, because lead times of 12–24 months are usually required for vaccine planning, production, testing, and release. In general for OPV, about 9–12 months are required for production, testing, and release of bulk vaccine, with an another 3–12 months required for formulation, filling, packaging, testing, and releasing of the finished product. Bulk manufacturers were therefore already required to make production decisions in 2013 for OPV2 supply in late 2015.

Lack of visibility of future vaccine demand due to the discordant timelines for planning between program and vaccine manufacturers created a major risk of losing tOPV production capacity should manufacturers decide to stop production of bulk OPV2 early to reduce potential financial risks. On the other hand, any delay in OPV2 cessation would require continuation—or restart—of tOPV production.

## REQUIREMENT FOR EARLY PLANNING AND CONTINUOUS COORDINATION WITH INDUSTRY IN SECURING MILESTONE

The October 2015 timeline for confirming tOPV cessation was indicated in early 2014. To minimize supply risk to the program, GPEI acknowledged the need to plan with industry as if cessation was moving ahead, while ensuring that contingency plans were in place in the event of a delay. In May 2014, GPEI conducted initial forecasts for the tOPV requirements, which were then communicated to bulk vaccine manufacturers during a high-level OPV2 withdrawal planning meeting. Key requests to vaccine manufacturers were the need for flexibility, opportunities for collaboration, maximizing capacity, and identification of options for contingencies in the event of delay. In addition to ensuring close coordination with manufacturers, GPEI partners organized regular reviews of the SIA calendar to improve visibility of vaccine requirements, allowing UNICEF to make timely increases in awards leading up to cessation to secure sufficient supply. 

Owing to the low and declining vaccine requirements projected at the time of the tender in 2012, in anticipation of interruption of wild poliovirus transmission by 2014, multiple additional awards were made to manufacturers of prequalified OPV—both tOPV and bOPV—throughout the period, increasing supply from 1.2 billion doses as projected in 2012 for 2014 to the maximum supply capacity of 1.9 billion doses, and similarly for 2015 from 1.0 to 1.6 billion doses. Despite projections of a declining market as provided in 2012, manufacturers have therefore seen OPV demand increase and plateau during the period, at an annual average of about 1.5 billion doses for the last 5 years.

After the October 2015 confirmation in by SAGE that cessation would take place in April 2016, UNICEF developed an algorithm for supply allocations to ensure fair and equal utilization of tOPV supply on contracts across manufacturers, to spread any demand and financial risk. Overall, there has been close collaboration between the GPEI and vaccine manufacturers of prequalified vaccines throughout the process leading to cessation, ensuring full transparency on program milestones and decision points, in recognition of the shortcoming that overall demand and timelines were not finally confirmed until October 2015.

## BALANCING OPPOSING RISKS OF VACCINE SHORTAGE AND EXCESS TRIVALENT OPV SUPPLY

Owing to intensive planned campaign activities during the low-transmission seasons in Q1 of 2015 and 2016, UNICEF required vaccine manufacturers to produce at full capacity, build up stocks, and keep these vaccines in the warehouses during Q4 of 2014 and 2015 for later usage. This provided challenges for vaccine manufacturers on 2 fronts: (1) the increasing stocks of vaccines across year end raised concerns of financial loss (particularly in 2015 owing to cessation), of shelf-life ticking and and reduced shelf-life resulting in potential nonacceptance of supplies by countries, and of accountability to senior management given the tying up of working capital; and (2) the freezing capacity for storing OPV was exhausted or close to being exhausted. One manufacturer stopped production temporarily after having explored alternative options to expand freezing capacity. This reduced the supply availability for the upcoming high peak season.

**Figure 2.**
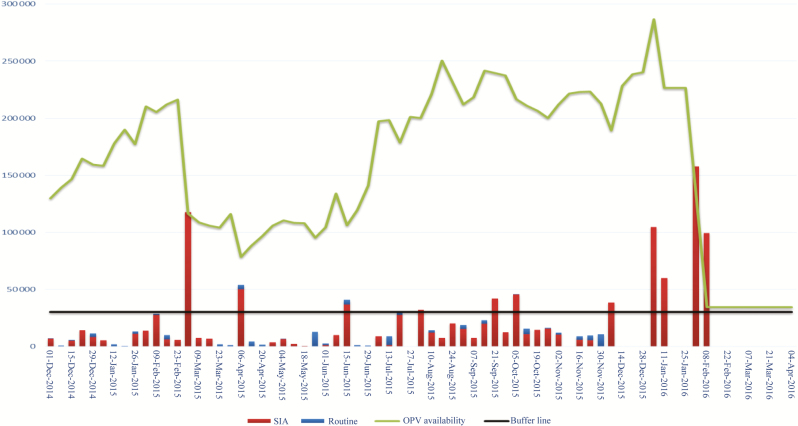
Projected cumulative supply balances as of October 2015 for April 2016. Abbreviations: OPV, oral poliovirus vaccine; SIA, supplementary immunization activity.

As presented to vaccine manufacturers during the Vaccine Industry Consultation in October 2015, a total of 280 million doses were projected to be required for carryover across year end to meet program requirements in Q1 of 2016; by the end of 2014, the projected carryover was about 200 million doses (Figure 2).The perception of risk increased with increasing stocks and led to a change in suppliers’ behavior, with 3 vaccine manufacturers requesting demand guarantees from UNICEF during Q4 of 2015 and Q1 of 2016 to mitigate financial risks, despite the fact that existing contractual frameworks were based on good-faith agreements. In each case, based on further negotiations between manufacturers and UNICEF, and owing to a long-term working relationship with all 3 manufacturers, vaccine manufacturers agreed to carry the risk based on UNICEF’s confirmation that most of the doses would be required and the residual stocks at the time of the switch would not be substantial.

On the program side, during the third quarter of 2015, concerns were also raised that supply through to cessation would not be sufficient, owing to increasing requirements for SIAs and unplanned immunization activities caused by outbreaks in some countries. The vaccine requirements were reassessed based on (1) country forecasts for routine requirements; (2) updates of the calendar of SIAs to mitigate identified country risks, based on models from multiple sources—the Centers for Disease Control and Prevention, the World Health Organization /Imperial College and Institute for Disease Modeling—as well as acute flaccid paralysis (AFP)-based susceptibility indicators, the proximity to recent polio cases, and other information, such as coverage of poliovirus vaccination (data presented by WHO during the WHO/UNICEF consultation with OPV and inactivated poliovirus vaccine manufacturers, 28 July 2016); and (3) a projection of vaccine requirements to address ongoing and future type 2 outbreaks and events, factoring in historic data from 2010 to 2015 after applying corrections for more sensitive case definitions (approved in mid-2015), with a target population based on outbreak response and vaccine wastage rates in the past 5 years. 

To ensure a continuous target buffer of about 40 million doses through cessation for any unplanned activities, after consultations with GPEI, UNICEF awarded an additional 70 million doses in October 2015, which were readily available in the market. The GPEI also requested UNICEF to ensure a physical stockpile of 8.8 million doses of tOPV to be available for immediate delivery in case of an outbreak. At that time, the GPEI also discussed and agreed in principle to coshare with vaccine manufacturers financial losses within a certain financial budget for contracted residual stocks of tOPV at the time of cessation, should manufacturers request such compensation.

## OUTCOME

The critical outcome to secure sufficient supply to meet the demand through tOPV cessation for programs procuring vaccines through UNICEF was fully achieved, while at the same time minimizing residual stocks with manufacturers. By April 2016, at the time of tOPV cessation, 64 tOPV million doses (5%) remained undelivered in manufacturers’ warehouses, of a total of 1.2 billion doses on contracts with UNICEF for delivery in 2015 and 2016. This corresponded to 24 million doses more than the minimum buffer of 40 million doses required by the GPEI. Utilization of quantities on contracts across 5 manufacturers ranged between 93% and 95%, ensuring a very high utilization of good-faith supply arrangements, as well as a fair and equal distribution of the financial burden related to residual stocks, when compared with the total number of tOPV doses delivered by each manufacturer in 2015 and 2016. On request from some manufacturers and on the provision of cost information, all manufacturers with residual stocks were offered a partial compensation, which was negotiated within the allocated budget. Four of 5 manufacturers requested compensation.

Beyond the residual stocks under UNICEF’s contracts, 6 vaccine manufacturers to UNICEF had tOPV stocks of about 100 million doses of released vaccines and 360 million doses of bulk OPV2 at the time of tOPV cessation. In addition, about 100 million doses of tOPV were withdrawn and destroyed at the country level across 155 OPV-using countries after cessation, bringing the total commercial value to about $36 million for about 264 million doses of tOPV residual stocks, estimated at UNICEF’s weighted average price of $.137 per dose.

From 2013, when the supply under the current procurement round started, through April 2016, when the tOPV market came to an end, no vaccine manufacturers exited the market, despite the increasing risk. On the contrary, one new manufacturer had its vaccine prequalified for the first time in 2013, and another regained prequalification of its vaccine after considerable investments in upgrading quality management systems, increasing the overall number of suppliers, which was considered important from a risk management perspective, and considerably increasing the supply capacity. This allowed UNICEF to meet the considerable increase in demand that materialized since 2012 owing to outbreaks.

UNICEF conducted a survey with all vaccine manufacturers of prequalified vaccines on the tOPV cessation, with 5 of 6 manufacturers responding. The survey was conducted to determine, from the manufacturers’ perspective, (1) what worked well related to the management of the tOPV cessation, (2) what did not work, (3) what improvements could be made for future cessation, and (4) the extent to which tOPV cessation was considered successful from a business perspective. Three of 5 manufacturers considered the outcome very successful, and 2 considered it somewhat successful. All manufacturers developed a strategy to manage the cessation, with different levels of senior management involvement. Two manufacturers started planning already in 2013, with cross-functional engagement in the planning process. Although the sample size is small, it seems likely that the earlier start of planning for the cessation, and hence a longer planning period, and wide engagement across multiple functions could increase the likelihood of achieving what is considered a successful outcome from a business perspective.

With regard to collaboration and communication, from suppliers’ perspective, UNICEF provided sufficient and frequent information about tOPV cessation, with opportunity for clarification as the situation changed. Most manufacturers confirmed that actual procurement was within an acceptable margin of deviation compared with the forecasts provided. Four of 5 manufacturers indicated that UNICEF’s forecast was timely for planning tOPV cessation. Only 1 manufacturer referred to the cosharing of financial risk of the residual stocks as an important measure for assessing outcome, but despite the compensation, this manufacturer rated the tOPV cessation as only somewhat successful.

**Figure 3.**
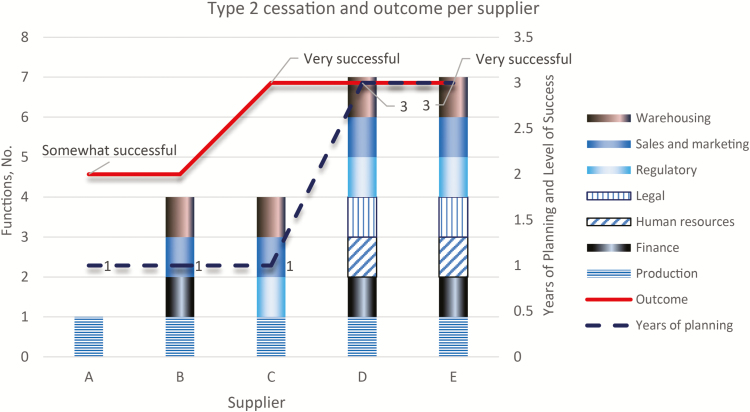
Years of planning and number of functions involved in the plans compared with the overall outcome of the switch as ranked by supplier.

Guidance from vaccine manufacturers for future cessation was that early communication would be required to allow integration into manufacturers’ strategies but also to involve manufacturers in early stages of decision making (Figure 3). A continuous close collaboration and coordination with GPEI and UNICEF was recommended, in order to review plans and solve issues as they occur. Manufacturers further cautioned about production and industrial investment timelines, and requested that production lead times should be considered when establishing timelines for confirmation of cessation.

## DISCUSSION AND LESSONS LEARNED

Some of the lessons learned from the cessation of the global supply market for tOPV are relevant for the upcoming global cessation of the OPV market but may also be relevant for other future planned cessation of other supply markets. In response to the OPV tender conducted in 2012, vaccine manufacturers did not require firm guaranties to mitigate perceived demand risks related to tOPV cessation. This may be for a number of reasons, including the history of the program’s setting dates for polio eradication that were not achieved or a lack of appreciation in 2012 of the demand risk in 2016, as well as the continuous increase in demand compared with original awards made in 2012. Another reason could be that while OPV2 would no longer be required after tOPV cessation, except for monovalent OPV2 for outbreak control, bulk types 1 and 3 OPV would still be required, and no major shifts were required from vaccine manufacturers, because the production, filling, packaging, testing, and releasing of bOPV continued at the same high level of resource requirements. 

With the full OPV withdrawal, the risk profile to vaccine manufacturers increases considerably, including the need to plan well in advance for permanent reallocation of resources, such as human resources, buildings, and equipment, as also indicated by vaccine manufacturers. Without full visibility of supply requirements and timing of cessation, there is a high risk that vaccine manufacturers will scale back production and plan their exit strategy out of sync with the needs of the GPEI. The ability to ensure access to sufficient supply through cessation will require continued close collaboration and coordination with vaccine manufacturers, ensuring that the timeline for OPV cessation fully incorporates and builds on production lead times. Even with these processes in place, market exits can be anticipated, owing to overall declining demand and the inability to keep all manufacturers in the market at a production scale that will allow affordable prices for the GPEI.

Manufacturers’ stocks toward cessation were closely monitored and managed under contracts with UNICEF, with utilization at about 95% across manufacturers, ending up with residual stocks of about 64 million doses across manufacturers. Although this level of supply constituted an acceptable buffer of tOPV to the GPEI and vaccine manufacturers at the time of cessation, it is likely that the GPEI, based on the recent outbreak of wild poliovirus type 1 in Nigeria in August 2016, will request an increase in buffer in case of unplanned requirements toward OPV cessation, thereby increasing the risks of residual stocks. Based on the recent increased awareness of demand risk and change in manufacturers’ behavior toward tOPV cessation, early indications from the GPEI about the willingness to coshare financial risks at the time of cessation may facilitate more affordable prices and prevent unwanted market exits.

Furthermore, it should be taken into consideration that there are physical limitations to the freezing storage capacity of vaccine manufacturers and no incentives to invest further owing to the upcoming cessation. To avoid peaks in demand requiring the building up of stocks—which contributes to the perception of increasing financial risks, but also provides physical challenges with storage and freezing capacity at manufacturer facilities—the GPEI could consider alternative strategies to ensure a more even distribution of demand, provided they do not affect programmatic outcomes, for example, continuing regular campaigns instead of intensified precessation immunity boosting campaigns immediately before cessation.

## CONCLUSIONS

A number of useful lessons have been learned that could be applied to improving supply management toward final OPV cessation. If carefully planned for and based on a close collaboration between program partners and manufacturers, the cessation of a supply market can be undertaken with a successful outcome for both parties. The need to consider industry production timelines when establishing timelines for the final OPV cessation is critical to ensure sufficient supply to achieve program outcomes while allowing manufacturers to undertake a managed scale-down of production to reduce risks.

Engagement with vaccine manufacturers, including during the planning stages, is required to ensure visibility on requirements at least 2 years in advance, considering production timelines; the early engagement from partners greatly facilitated the success from a business perspective and is assumed to potentially minimize financial loss across manufacturers. Ensuring transparency on the milestones and timelines toward cessation despite epidemiological unpredictability facilitated a good understanding with vaccine manufacturers.

The perception of financial risk among vaccine manufacturers can be expected to increase toward final OPV cessation, with risks probably perceived to be considerably higher than those related to tOPV cessation. To avoid premature market exits, the GPEI should consider deciding on and communicating a suitable mechanism for cosharing financial risks or other financial arrangement for the outer years, to ensure a reliable and sustainable supply of OPV up through cessation without risking shortages of supply or compromising the ability to achieve polio eradication.

## Notes

***Supplement sponsorship.*** This work is part of a supplement coordinated by the Task Force for Global Health with funding provided by The Bill and Melinda Gates Foundation and the Centers for Disease Control and Prevention.

***Potential conflicts of interest.*** All authors: no reported conflicts. All authors have submitted the ICMJE Form for Potential Conflicts of Interest. Conflicts that the editors consider relevant to the content of the manuscript have been disclosed.
